# An Account of Diseases in an Eastern District of London, from Oct. 20 to Nov. 20, 1810

**Published:** 1810-12

**Authors:** 


### *An Account of Diseases in an Eastern District of London, from Oct. 20 to Nov. 20, 1810.*

---

| | | | |
|---|---|---|---|
| ACUTE DISEASES. | | Dysmenorrhœa - | 2 |
| Pneumonia - - | 3 | Leucorrhœa - | 6 |
| Cynanche Tonsillaris - | 4 | Dysuria - - | 4 |
| Variola - - - | 9 | Hypochondriasis - - | 1 |
| Morbilli - - - | 8 | Chorea - - | 1 |
| Rheumatismus Acutus - | 3 | Rheumatismus Chronicus | 6 |
| CHRONIC DISEASES. | | PUERPERAL DISEASES. | |
| Tussis - - - | 10 | Menorrhagia Lochialis | 3 |
| Dyspnœa - - | 8 | Ephemera - - | 2 |
| Tussis cum Dyspnœa - | 7 | Dolores post Partum - | 5 |
| Phthisis Pulmonalis - | 2 | Dysuria - - | 4 |
| Hydrothorax - - | 3 | INFANTILE DISEASES. | |
| Asthma - - | 1 | Convulsio - - - | 1 |
| Cholera - - - | 2 | Rachitis - - - | 2 |
| Diarrhœa - - | 4 | Tabes Mesenterica - | 3 |
| Enterodynia - - | 5 | Vermes - - - | 4 |
| Menorrhagia - - | 3 | Dentitio - - - | 2 |

---

The Small Pox has lately prevailed to a very considerable degree. The infection has been propagated very diffusely: whole families of children have been affected by it, and, as the consequence of an intercourse kept up in the neighbourhood of some confined situations, the disease has spread to a very great extent. Most of the cases referred to in the list were attended with very unpleasant and dangerous symptoms, the number of pustules was large, and the disease of the confluent species. In one of the cases a fatal termination took place.

In many of the patients who were visited by this loathsome disease it has been succeeded by some morbid affection of the eyes. A considerable redness and fullness of the vessels of the tunica conjuctiva, attended with an intolerance of light and a great flow of tears, indicated an inflamed state of the organ.

The Meazles have likewise been very frequent of late, and, as in the Small Pox, have been accompanied with a train of unpleasant symptoms. Pneumonic affections have been almost the constant attendants upon the disorder, and these have continued to harass the patient for a considerable time after the eruptive disease has gone through its usual stages.

